# Role of *beta-(1→3)(1→6)-D-glucan* derived from yeast on natural killer (NK) cells and breast cancer cell lines in 2D and 3D cultures

**DOI:** 10.1186/s12885-024-11979-3

**Published:** 2024-03-14

**Authors:** Abdelhadi Boulifa, Martin J. Raftery, Alexander Sebastian Franzén, Clarissa Radecke, Sebastian Stintzing, Jens-Uwe Blohmer, Gabriele Pecher

**Affiliations:** 1 Berlin Institute of Health at Charité – Universitätsmedizin Berlin, Charitéplatz 1, Berlin, 10117 Germany; 2 Competence Center of Immuno-Oncology and Translational Cell Therapy (KITZ), Department of Hematology, Oncology and Tumor Immunology, CCM, Charité–Universitätsmedizin Berlin, Corporate Member of Freie Universität Berlin and Humboldt-Universität zu Berlin, Charitéplatz 1, Berlin, 10117 Germany; 3 Department of Gynecology with Breast Center Charité - Universitätsmedizin Berlin, Corporate Member of Freie Universität Berlin and Humboldt-Universität zu Berlin, Charitéplatz 1, Berlin, 10117 Germany

**Keywords:** Beta-glucan, Breast cancer, NK cell, Anticancer, Spheroids

## Abstract

**Background:**

*Beta-(1,3)(1,6)-D-glucan* is a complex polysaccharide, which is found in the cell wall of various fungi, yeasts, bacteria, algae, barley, and oats and has immunomodulatory, anticancer and antiviral effects. In the present study, we investigated the effect of *beta-(1,3)(1,6)-D-glucan* derived from yeast on the proliferation of primary NK cells and breast cancer cell lines in 2D and 3D models, and on the cytotoxicity of primary NK cells against breast cancer cell lines in 2D and 3D models.

**Methods:**

In this study, we investigated the effects of different concentrations of yeast-derived *beta-(1→3)(1→6)-D-glucan* on the proliferation and cytotoxicity of human NK cells and breast cancer cell lines in 2D and 3D models using the XTT cell proliferation assay and the CellTiter-Glo® 2.0 assay to determine the cytotoxicity of human NK cells on breast cancer cell lines in 2D and 3D models.

**Results:**

We found that the co-incubation of NK cells with beta-glucan in the absence of IL2 at 48 h significantly increased the proliferation of NK cells, whereas the co-incubation of NK cells with beta-glucan in the presence of IL2 (70 U/ml) increased the proliferation of NK cells but not significantly. Moreover, beta-glucan significantly inhibited the proliferation of breast cancer cell lines in 2D model and induced a weak, non-significant growth inhibitory effect on breast cancer multicellular tumor spheroids (3D). In addition, the cytotoxicity of NK cells against breast cancer cell lines was examined in 2D and 3D models, and beta-glucan significantly increased the cytotoxicity of NK cells against MCF-7 (in 2D).

**Conclusions:**

Yeast derived *beta-(1,3)(1,6)-D-glucan* could contribute to the treatment of cancer by enhancing NK cell immune response as well as contributing to inhibition of breast cancer cell growth.

**Supplementary Information:**

The online version contains supplementary material available at 10.1186/s12885-024-11979-3.

## Introduction

Cancer is one of the most common causes of death in the world. In 2020, an estimated 19.3 million people worldwide were newly diagnosed and nearly 10 million died from cancer [1]. According to the International Agency for Research on Cancer (IARC), breast cancer (BC) has recently become the most commonly diagnosed cancer worldwide with an estimated 2.3 million new cases (11.7%) followed by lung cancer (11.4%), colorectal cancer (10%), prostate cancer (7.3%), and stomach cancer (5.6%) [1]. The development of breast cancer is influenced by several factors such as hereditary genetics, lifestyle, gender, and age, the latter being the most important risk factor [2].

Currently, there are several methods for the treatment of breast cancer. Treatment efficacy for patients with advanced breast cancer is still unsatisfactory despite the use of various treatment modalities such as immunotherapy, chemotherapy, hormone therapy, targeted therapy, radiotherapy, endocrine therapy, and surgical resection [3, 4], which is considered the most recommended approach to treat primary breast cancer [5]. Postoperative prognosis is disappointing due to the appearance of residual tumor cells, leading to high mortality [6, 7]. Moreover, cancer therapies are often associated with side effects and high resistance, which is why a new effective supportive therapy is an essential part of the treatment concept of patients with breast cancer, such as the use of natural products derived from plants, animals, fungi and other living microorganisms, which can ameliorate cancer progression and the side effects of various tumor therapies [8, 9].

Beta-glucans are polysaccharides of *D-glucose* monomers linked by glycosidic bonds. They are widely distributed in the cell walls of yeasts, fungi, some bacteria, algae, and cereals (oats and barley) [10, 11]. The different forms of beta-glucan identified from various sources vary in their molecular weight, length, shape, beta-linkage, and macromolecular structure either by different branching and linkage or by attachment of other types of molecules such as proteins [12, 13]. These differences in the shape and structure of glucans are an important factor influencing the expression of their biological activities. The *beta-1,3-D-glucose* unit of beta-glucan is the linear core structure and the most abundant form, for example the yeast and fungal beta-glucan contain a *beta-(1,3) backbone* with *(1,6)-side branches* and have antitumor, anti-inflammatory and antioxidant effects, the bacterial beta-glucan is structured with a *beta-(1,3) bond* and has an immunomodulatory effect, and cereal beta-glucan is linked to the *beta-(1,3)-* and *beta-(1,4)-backbones* with hypoglycemic effect and biological anti-cholesterolemic activity [14–17]. These variations lead to different biological activities which are found as functional and bioactive food constituents [18].

In vitro, animal and human clinical studies have demonstrated the immunomodulatory activities of beta-glucan, highlighting its ability to protect against infection and enhance vaccine immunogenicity, as well as its antitumor activity [19, 20]. Mushroom-beta-glucan, along with other formulations, has been used as an adjuvant in both cancer prophylaxis and therapy, as well as a single agent for immunomodulatory purposes, and is therefore referred to as an immunoadjuvant [21].

Beta-Glucan possess several membrane-bound receptors that can be recognized by specific receptors on cell membranes of the immune system, such as Dectin-1 [22, 23], Complement Receptor 3 (CR3), CD11b / CD18 [24], scavenger receptors [25], Toll-like receptors (TLR) [26], and lactosylceramide [27], which were involved in beta-glucan signaling as pathogen-associated molecular patterns (PAMP). Fungal beta-glucan can function as PAMPs or MAMPs (Microbe-Associated Molecular Patterns) by triggering host immune responses after contact. In particular, beta-glucan can bind to defense cells via these receptors and stimulate their activation, differentiation, and production of ROS (Reactive oxygen species) and cytokines in neutrophils, dendritic cells, macrophages, T-lymphocytes, B-lymphocytes, and NK cells to enhance microbial killing and immune response against pathogens and tumors [28, 29].

The aim of the present study was to evaluate the therapeutic potential of beta-glucan on stimulating primary NK cell activity and antitumor activities on breast cancer cell line in a 2D and 3D culture system.

## Materials and methods

### Cell lines and cell culture

All work with cell cultures was always performed under sterile conditions. All cell types were incubated in a CO_2_ incubator at 37 °C, 5% CO_2_, 95% humidity, and regularly tested for mycoplasma (MycoAlert Lonza). The breast cancer cell lines MCF-7, T47D, SK-BR-3, HCC1937, obtained from DSMZ-German Collection of Microorganisms and Cell Cultures GmbH, and the MRC5 fibroblasts, obtained from LGC Standards GmbH, were maintained in Roswell Park Memorial Institute (RPMI) 1640 medium (Gibco) supplemented with 10% fetal bovine serum (FBS; Gibco), 200mM L-Glutamine and 1% penicillin/streptomycin (Pen/Strep; Gibco). The breast cancer cell lines MDA-MB-231 was cultured in Dulbecco’s modified Eagle’s medium (DMEM; Gibco) supplemented with 10% fetal bovine serum (FBS; Gibco), 200mM L-Glutamine and 1% penicillin/streptomycin (Pen/Strep; Gibco).

MCF-7 cells were isolated from a 69-year-old woman’s pleural effusion who had metastatic disease. The name MCF-7 comes from the Michigan Cancer Foundation, where it was founded in 1973 [30]. They are estrogen (E2)-sensitive cells and depend on E2 to proliferate [31]. They express high levels of estrogen receptor alpha (ERα) transcripts but low levels of estrogen receptor beta (ERβ) [32].

The breast cancer cell line HCC1937 (Harmon Cancer Center) was derived from a grade III invasive ductal primary tumor in a 24-year-old breast cancer patient with a BRCA1 germline mutation. During histological examination of the primary tumor, large vacuoles were observed in many cells, suggesting a secretory variant of invasive intraductal carcinoma [33, 34].

T47D are epithelial cells isolated from the pleural fluid of a 54-year-old female patient with invasive ductal carcinoma of the breast [35]. They represent luminal A condition, a subtype of breast cancer [36].

The SK-BR-3 cell line (Sloan-Kettering Breast Cancer Cell Line 3) was isolated in 1970 from pleural effusion cells of a 43-year-old adenocarcinoma patient and is named after the Memorial-Sloan Kettering Cancer Center [37, 38]. It is a HER2 + breast cancer cell line that has no hormone receptors for ER or PR [39].

The fibroblast and breast cancer cell lines were regularly passaged once per week. For passaging of adherent cells the method of cell dissociation with trypsin was used. After passage, the cell pellet was resuspended in fresh medium and cell number was determined using Trypan blue exclusion assay.

### Reagents

The aqueous preparation of *beta-(1–3)(1–6)-D-glucan* (20 mg/ml), derived from the inner cell wall of Saccharomyces cerevisiae, was provided by Biotec Pharmacon (Tromsø, Norway) and was dissolved in PBS. The glucan preparation contained a fraction of aggregated glucan polymers, and its endotoxin level was < 0.05 EU/ml.

### NK cell isolation

Primary NK cells were isolated from buffy coats (DRK, German Red Cross; Germany) using density gradient centrifugation. First, PBMCs (Peripheral Blood Mononuclear Cells) were isolated from the buffy coats, followed by isolation of NK cells from the PBMCs using the human NK cell isolation kit (Miltenyi Biotec) according to the manufacturer’s protocol. The isolated NKs were tested for purity by flow cytometry and further cultured in 50% AIM V™ Medium (Gibco) and 50% NK MACS medium (Miltenyi) supplemented with 5% AB serum (Merck), 500 U/mL IL-2 (ImmunoTools), 140 U/mL IL-15 (ImmunoTools), 1% NK MACS Supplement and 1% penicillin/streptomycin (Pen/Strep; Gibco).

### Preparation of 3D-cell culture (multicellular tumor spheroids)

Breast cancer cell lines MCF-7, HCC1937, T47D and the fibroblast MRC5 were used to prepare the spheroids. First, the 96-well round bottom plate was pre-coated with anti-adherence rinsing solution (Stemcell Technologies). Then, cells were counted, seeded on the pre-coated 96-well plate at 3 × 10^3^ cells/well in triplicate, and centrifuged at 100 g for 5 min. This allowed the cells to collect at the bottom and grow into compact spheroids over 72 h. The incubation was performed in an incubator at 37 °C, 5% CO_2_ and 95% humidity. To better model the breast cancer cell line MCF-7 and obtain a uniform MCTS (Multicellular Tumor Spheroids), we added MRC5 fibroblasts to MCF-7 at a ratio of 1:1 (MCF7:MRC5).

### 2,3-Bis-(2-Methoxy-4-Nitro-5-Sulfophenyl)-2 H-Tetrazolium-5-Carboxanilide (XTT) cell proliferation assay

**Fig. 1:**
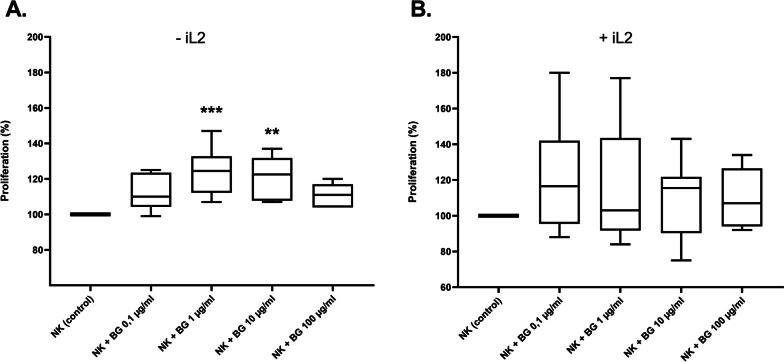
Effect of *beta-(1→3)(1→6)-glucan *from yeast on the proliferation of the primary NK cells. The figure shows the percentage variation of NK proliferation after 48 h incubation of (**A**) NK cells with different β-glucan concentrations (0.1–1 − 10 and 100 µg/ml) without IL2 and (**B**) NK cells with different β-glucan concentrations (0.1–1 − 10 and 100 µg/ml) with IL2 (70 U/ml) compared to untreated NK cells (control). The proliferation was measured after 4 h incubation of untreated- and with β-glucan treated NK cells using XTT by 450 nm. The results were presented in three independent experiments. Statistical significance was determined by one-way ANOVA. * *p* < 0.05; ** *p* < 0.01; *** *p* < 0.001; **** *p* < 0.0001

NK cells were counted, washed with PBS to eliminate all cytokines, and resuspended in RPMI-1640 supplemented with 10% FBS (Gibco), 200 mM L-glutamine, and 1% Pen/Strep (Gibco), once with 70 U/ml IL2 (ImmunoTools) (Fig. 1B) and once without IL2 (Fig. 1A). Next, the NK cells were seeded with different beta-glucan concentrations (0.1–1 − 10 and 100 µg/ml)) at a density of 1.5 × 10^5^ cells/well in triplicate. Then, the NK cells were incubated in an incubator at 37 °C and 5% CO_2_ for 48 h.

Control wells were treated with culture media only (beta-glucan concentration = 0). After an incubation period of 48 h, cells were treated with XTT by adding the mixture of XTT-labeling reagent (50 µl/well) and electron coupling reagent (1 µl/well), the plate was then mixed gently and incubated at 37 °C in a CO_2_ incubator for 4 h. After incubation with XTT, the plate was gently shaken to distribute the dye evenly and absorbance was measured at 450 nm in an ELISA reader to determine the proliferation of NK cells after treatment with beta-glucan compared to untreated NK cells.

Breast cancer cell lines (2D) were passaged, counted, and seeded in 1 ml RPMI-1640 for MCF-7, T47D, SK-BR-3 and HCC1937, and in 1 ml DMEM for MDA-MB-231, supplemented with 10% FBS (Gibco), 200 mM L-glutamine and 1% Pen/Strep (Gibco) in duplicates of a 24-well plate (Sarstedt). The breast cell lines (2D) were then incubated with different concentrations of beta-glucan (0.1–1 − 10 and 100 µg/ml) at 37 °C in a CO_2_ incubator for 24 h, 48 and 72 h. After incubation, the supernatants were removed, 150 µl of fresh medium was added and treated with XTT according to the manufacturer’s protocol to determine the proliferation of breast cancer cell lines (2D) after treatment with beta-glucan compared to untreated cells (beta-glucan concentration = 0).

The Multicellular Tumor Spheroids of breast cancer cell lines were prepared as described above for 3 days, then fresh medium with different beta-glucan concentrations was added to spheroids and further cultured in an CO_2_ incubator at 37 °C. After 24 h, 48 and 72 h, spheroids proliferation was measured after treatment with beta-glucan using XTT according to the manufacturer’s protocol.

### Cytotoxicity assays

The CellTiter-Glo® 2.0 Assay (Promega) was used to measure cytotoxicity following the manufacturers protocol. Briefly, cells were incubated at the indicated E:T ratios with different beta-glucan concentrations (0.1–1 − 10 and 100 µg/ml). After incubation (48 h for 2D cultures and 72 h for 3D cultures), CellTiter-Glo® reagent was added according to the manufacturer’s protocol and subsequently measured using a Tristar 3 multimode plate reader (Berthold Technologies). The addition of CellTiter-Glo® reagent resulted in cell lysis and the generation of a luminescence signal. The generated luminescence signal represents the amount of ATP present, which corresponds to the amount of cells present in the culture. The cytotoxicity of NK cells was calculated from the ATP released from the target and effector cells using the following formula:

$$\mathrm{Cytotoxicity}\;(\%)=\left(100-\frac{\mathit S\mathit a\mathit m\mathit p\mathit l\mathit e\mathit\;\mathit r\mathit e\mathit l\mathit e\mathit a\mathit s\mathit e\mathit-\mathit E\mathit f\mathit f\mathit e\mathit c\mathit t\mathit o\mathit r\mathit\;\mathit r\mathit e\mathit l\mathit e\mathit a\mathit s\mathit e\mathit\;}{\mathit T\mathit a\mathit r\mathit g\mathit e\mathit t\mathit\;\mathit r\mathit e\mathit l\mathit e\mathit a\mathit s\mathit e}\right)\times100$$

### Statistical analysis

Statistical analysis was performed using Prism (v. 8.4.2, Graphpad). The data were compared using the One-way and Two-way ANOVA test. Statistical significances are marked *p* < 0.05 = *, *p* < 0.01 = **, *p* < 0.001 = ***, *p* < 0.0001 = **** and non-significance is marked with ns.

## Results

To investigate whether particulate beta-glucan derived from yeast could stimulate proliferation of primary NK cells, we co-incubated NK cells isolated from PBMC (Peripheral Blood Mononuclear Cells) with different beta-glucan concentrations (0.1–1 − 10 and 100 µg/ml) and measured proliferation after 48 h using XTT (Fig. 1). The co-incubation of primary NK cells with beta-glucan in the absence of IL-2 after 48 h showed that beta-glucan significantly increased the proliferation of NK cells to 24% at the concentration of 1 µg/ml and to 21% at the concentration of 10 µg/ml (Fig. 1A), while the co-incubation of NK cells with beta-glucan in the presence of IL2 (70 U/ml) also showed that beta-glucan increased the proliferation of NK cells to 39% at the concentration of 0.1 µg/ml and to 33% at the concentration of 1 µg/ml (Fig. 1B).

**Fig. 2:**
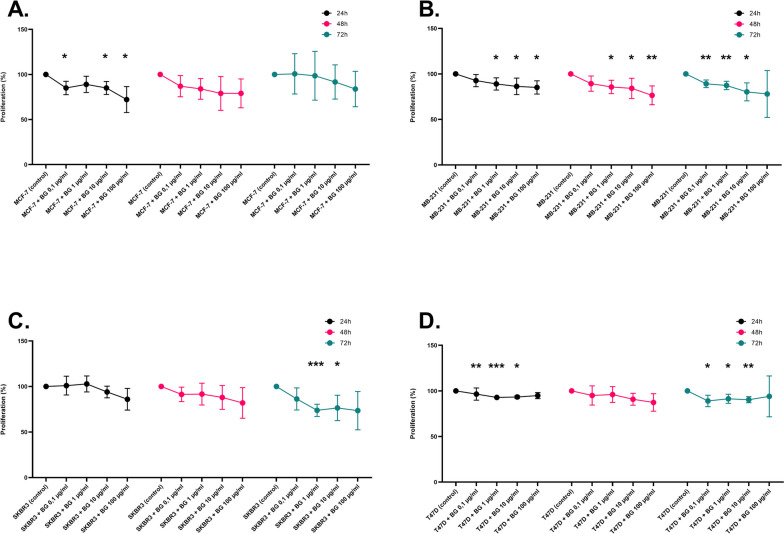
Effect of *beta-(1→3)(1→6)-glucan *from yeast on the proliferation of the breast cancer cell lines (2D). The figure shows the percent variation of proliferation of (**A**) MCF-7, (**B**) MDA-MB-231, (**C**) SK-BR-3, and (**D**) T47D with different β-glucan concentrations (0.1–1 − 10 and 100 µg/ml) after 24 h, 48 h, and 72 h incubation compared with untreated cells (control). The proliferation was measured after 4 h incubation of untreated- and with β-glucan treated breast cancer cells using XTT by 450 nm. The results were presented in three independent experiments. Statistical significance was deter-mined by two-way ANOVA. * *p* < 0.05; ** *p* < 0.01; *** *p* < 0.001; **** *p* < 0.0001

Yeast-derived *beta-(1→3)(1→6)-glucan* was used in this study to investigate the effect of beta-glucan on the proliferation of different breast cancer cell lines (in 2D model) after 24 h, 48 and 72 h. From Fig. 2A, it can be observed that the highest concentration of beta-glucan (100 µg/ml) showed the greatest effect on the proliferation of MCF-7 by significantly inhibiting the proliferation of MCF-7 by 28% after 24 h. After 48 h and 72 h, the beta-glucan concentration of 100 µg/ml also decreased the proliferation of MCF-7 by 21% and 16% respectively. In the case of MDA-MB-231 (Fig. 2B), yeast-derived *beta-(1→3)(1→6)-glucan* was able to inhibit the proliferation significantly after 24 h as well as 48 and 72 h. The highest inhibition of the proliferation was at the beta-glucan concentration of 100 µg/ml significantly by 15% after 24 h and 23% after 48 h. After 72 h, the proliferation was inhibited by 22% at the beta-glucan concentration of 100 µg/ml. The lowest inhibition was between 7% and 11% significantly after 72 h at the beta-glucan concentration of 0.1 µg/ml. Furthermore, the co-incubation of SK-BR-3 with yeast-derived *beta-(1→3)(1→6)-glucan* (Fig. 2C) showed that beta-glucan inhibited the proliferation of SK-BR-3 after 24 h, 48 h and significantly after 72 h. Here, it can be seen that at beta-glucan concentration of 1 and 10 µg/ml, the proliferation of SK-BR-3 showed a significant inhibition after 72 h, which was a decrease in proliferation by 26% and 23% consecutively. At the beta-glucan concentration of 100 µg/ml and after 72 h, the proliferation of SK-BR-3 decreased by 26% compared to the control. To this, beta-glucan showed no effect on SK-BR-3 after 24 h at the concentrations of 0.1 and 1 µg/ml, whereas at the concentration of 1 µg/ml, the proliferation of SK-BR-3 was slightly increased (by 3%) compared with the control. The same results were found for T47D (Fig. 2D) in that beta-glucan inhibited the proliferation of T47D also after 24 h, 48 h, and 72 h. After 24 h as well as 72 h, beta-glucan significantly inhibited the proliferation of T47D ranging from 7 to 11%. Furthermore, beta-glucan decreased the proliferation of T47D after 48 h up to 13% at a beta-glucan concentration of 100 µg/ml.

**Fig. 3:**
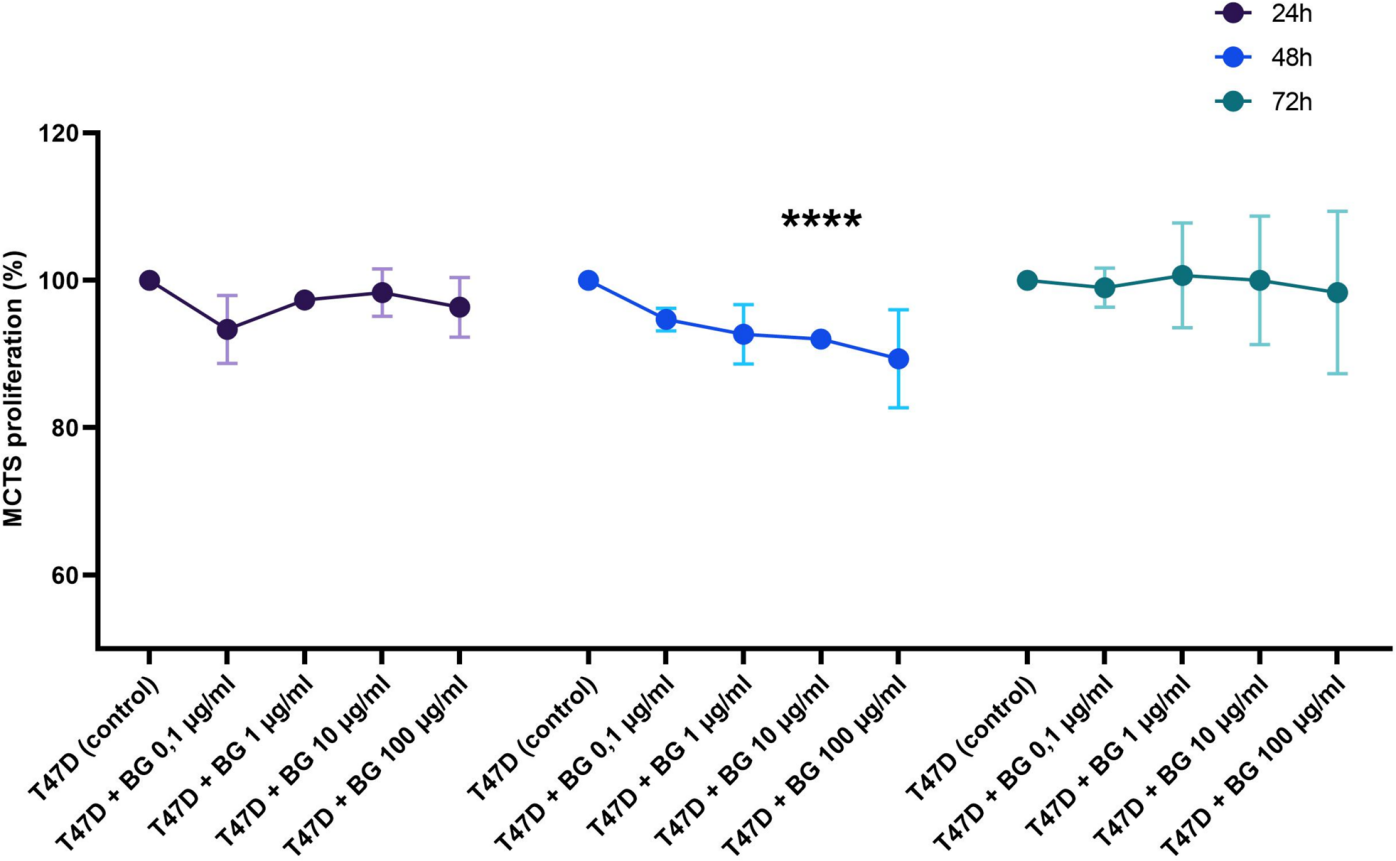
Effect of *beta-(1→3)(1→6)-glucan* from yeast on the proliferation of the breast cancer Multicellular Tumor Spheroids (MCTS) (3D). The figure shows the percent variation of proliferation of T47D spheroids with different β-glucan concentrations (0.1–1 − 10 and 100 µg/ml) after 24 h, 48 h, and 72 h incubation compared with untreated spheroids (control). The proliferation was measured after 4 h incubation of untreated- and with β-glucan treated breast cancer spheroids using XTT by 450 nm. The results were presented in three independent experiments. Statistical significance was determined by two-way ANOVA. * *p* < 0.05; ** *p* < 0.01; *** *p* < 0.001; **** *p* < 0.0001

In the present study, the MCTS from breast cancer cell lines was established and then incubated with yeast-derived *beta-(1→3)(1→6)-glucan* for 24 h, 48 and 72 h together to investigate how the effect of yeast-derived beta-glucan is on the MCTS from breast cancer cell lines. The Fig. 3 showed that yeast-derived *beta-(1→3)(1→6)-glucan* had small effect on the MCTS from breast cancer cell lines compared to breast cancer cell lines in 2D. It was shown that beta-glucan had a slight effect on the proliferation of MCTS of MCF-7:MRC5 after 24 h, 48 h as well as 72 h, by bringing down the proliferation up to 7% (data not shown). Furthermore, beta-glucan also showed slight effect on the proliferation of MCTS of HCC1937 in that beta-glucan brought the proliferation down to 8% after 24 and 48 h. After 72 h, beta-glucan showed no effect on proliferation except at the concentration of 100 µg/ml where the proliferation increased by about 8% (data not shown). In the case of MCTS of T47D, also the proliferation was inhibited by yeast-derived *beta-(1→3)(1→6)-glucan* up to 11% after 24 and 48 h. After 72 h, however, beta-glucan showed no effect on the proliferation of the MCTS of T47D (Fig. 3).

**Fig. 4:**
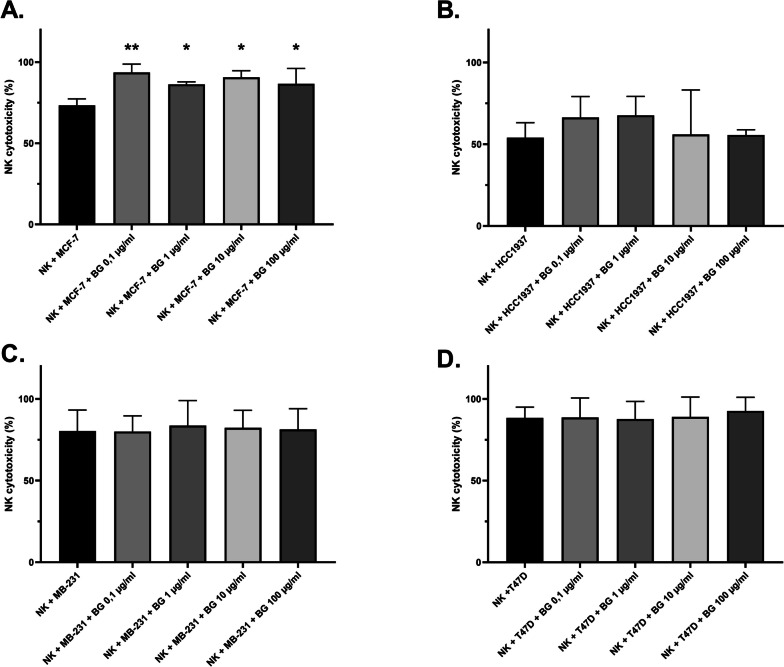
Effect of *beta-(1→3)(1→6)-glucan *from yeast on the cytotoxicity of NK cells against breast cancer cell lines (2D). The figure shows the percent variation of cytotoxicity of NK cells against (**A**) MCF-7, (**B**) HCC1937, (**C**) MDA-MB-231, and (**D**) T47D with different β-glucan concentrations (0.1–1 − 10 and 100 µg/ml) after 48 h incubation compared with untreated cells (control) at an E:T ratio of 3:1. The cytotoxicity was measured from the ATP released from the target and effector cells using CellTiter-Glo® 2.0 Assay. The results were presented in three independent experiments. Statistical significance was determined by one-way ANOVA. * *p* < 0.05; ** *p* < 0.01; *** *p* < 0.001; **** *p* < 0.0001

To test the major function of NK cells using yeast-derived *beta-(1→3)(1→6)-glucan* in the context of cellular immune defense in killing virus- and tumor-infected cells, yeast-derived *beta-(1→3)(1→6)-glucan* was incubated together with primary NK cells and breast cancer cell lines (in 2D Model) to investigate the cytotoxicity of NK cells in the presence of yeast-derived *beta-(1→3)(1→6)-glucan* (Fig. 4). As shown in Fig. 4A, beta-glucan could significantly induce the cytotoxicity of NK cells. The cytotoxicity of NK cells against MCF-7 increased significantly to 94% after 48 h in the ratio [3:1] [NK:MCF-7] at a beta-glucan concentration of 0.1 µg/ml compared with the control (73%). At beta-glucan concentrations of 1, 10, and 100 µg/ml, NK cell cytotoxicity ranged from 86 to 91% (Fig. 4A). The same conditions were used to examine the cytotoxicity of NK cells against HCC1937. Beta-glucan also showed an effect on the cytotoxicity of NK cells against HCC1937 after 48 h in the ratio of [3:1] [NK:HCC1937] and was 66% and 68% at the beta-glucan concentration of 0.1 and 1 µg/ml, respectively, compared with the control (54%). At beta-glucan concentration of 10 and 100 µg/ml, the cytotoxicity of NK cells against HCC1937 was 56% (Fig. 4B). However, beta-glucan showed small effect on the cytotoxicity of NK cells against MDA-MB-231 (Fig. 4C) and T47D (Fig. 4D) after 48 h in the ratio of [3:1], where beta-glucan increased the cytotoxicity of NK cells around 5% more compared to control.

Furthermore, the multicellular breast cancer tumor spheroids (3D in vitro models) were co-incubated in the present study with primary NK cells and *beta-(1→3)(1→6)-glucan* from yeast (at different concentrations) at a ratio of [3:1] [NK:MCTS] (Supplementary Fig. 1) to investigate the effect of *beta-(1→3)(1→6)-glucan* from yeast on the cytotoxicity of multicellular tumor spheroids from breast cancer (3D model). After 72 h, beta-glucan showed an effect on the cytotoxicity of NK cells against the co-culture of MCF-7:MRC5 [1:1] spheroids at the two highest beta-glucan concentrations, increasing cytotoxicity by 8% at 10 µg/ml and 14% at 100 µg/ml compared to the control. However, at the remaining beta-glucan concentrations, beta-glucan showed no effect on the cytotoxicity of NK cells (Supplementary Fig. 1A). Moreover, for the same conditions, beta-glucan showed small effect on the cytotoxicity of NK cells against the HCC1937 (Supplementary Fig. 1B) and T47D (Supplementary Fig. 1C) spheroids which were 3% more compared to the control.

## Discussion

Beta-glucan are immune-stimulatory substances that have a significant role in the therapy of cancer and infectious diseases. Yeast-derived beta-glucan can initiate the inflammatory process, activate the immune response, improve resistance to infection, and inhibit cancer development [40].

Previous studies have repeatedly shown that the addition of *beta-(1→3)(1→6)-glucan* from yeast or fungi was able to activate NK cells in cancerous mice [41] as well as in healthy adults [42] and inhibited a decrease in NK cell activity and cell number in the recovery phase after intense exercise [43]. In the present study, we could confirm that *beta-(1→3)(1→6)-glucan* from yeast has a stimulatory effect on the proliferation of primary NK cells after 48 h incubation with and without IL2 (Fig. 1) using XTT assay to assess mitochondrial activity and cell viability. Beta-glucan can trigger systemic immune-activation in fungal infections to interact with specific receptors to release the production of proinflammatory cytokines [44]. The activation of immune cells is thought to be due to the binding of beta-glucan to dectin-1 on immune cells, which in turn activate T cells, nuclear factor kappa B (NF-kB), and the MAP kinase (MAPK, mitogen activated protein kinase) signaling pathway by triggering the production of various cytokines [45, 46]. Studies have also shown that beta-glucan leads to the activation of monocytes and NK cells, and the production of IL-6 and IL-8 via dectin-1 and NKp30 respectively [47, 48]. This result was recently confirmed by Mehaj, V et al. [49].

Based on a study investigating over 60 different beta-glucans, Vetvicka and Vetvickova (2018, 2020) [50, 51] reported that beta-glucans showed significant anticancer, anti-infective, and immune-stimulatory activities. Moreover, several mushroom beta-glucans were commercialized as food supplements and subjected to clinical trials as cancer treatment adjuvants with inspiring results [52]. Furthermore, Dekker and Barbosa-Dekker also previously reported several biological activities of *beta-(1→3)(1→6)-glucan*. These include in vitro antiproliferative and proapoptotic properties in various tumor cell lines including human breast carcinoma (MCF-7) [53].

We were able to confirm these results in the present study by showing that *beta-(1→3)(1→6)-glucan* from yeast inhibited the proliferation of four breast cancer cell lines (MCF-7, SK-BR-3, MDA-MB-231, T47D) (Fig. 2). Various studies have already shown that beta-glucan has an anticancer effect on several cancer types [54–56] including breast cancer [57, 58] using 2D cell culture.

Breast cancer cell lines are frequently used to study the pathobiology of breast cancer and to screen and characterize new therapies. The use of different cell lines allows a better understanding of the diversity and heterogeneity of breast cancer [59, 60]. We chose breast cancer cell lines with different origins and agressiveness reflecting the association between loss of progesterone and estrogen receptors and poor therepeutic prognosis. The use of different cell lines makes it possible to test the efficacy of drugs in a broader context. For example, Herceptin has been found to predict therapeutic response in several HER2 + cell lines such as SK-BR-3 [61].

In order to successfully reproduce different aspects of the tumor microenvironment (TME), 3D cell culture such as multicellular tumor spheroids (MCTS) can be used as a reliable alternative [62, 63] to better evaluate the capacity of drugs to invade tumor tissues [64].

Pattern recognition receptors expressed on immune cells including macrophages, dendritic cells, neutrophils, and lymphocytes have been shown to be activated by beta-glucan [65, 66]. Several in vitro studies have shown that beta-glucan from yeasts, fungi, or cereals can enhance the function of human primary immune cells and elicit strong immune responses through their recognition of various PRRs (Pattern Recognition Receptors), particularly dectin-1 and complement receptor 3 (CR3) [67]. Therefore, it has been reported that the increasing of NK cell activity by beta-glucan plays an important role in immune potency in in vitro and in vivo studies [68, 69]. In addition, the effects of fungal or yeast beta-glucan on primary NK cells have shown that these polysaccharides significantly enhance NK cell cytotoxicity by stimulating the production of interferon-gamma and perforin and increasing the expression of the activating receptor NKp30 [70]. These cytotoxic effects are mediated by upregulation of NKG2D and IFN and enhanced in the presence of IL-2, while activation of cytokine production is associated with upregulation of KIR2DL genes [71]. It was confirmed in the present study that *beta-(1→3)(1→6)-glucan* from yeast markedly increased the cytotoxicity of NK cells against MCF-7 and HCC1937, however, the effect of beta-glucan on the cytotoxicity of NK cells against MDA-MB-231 and T47D was small (Fig. 4) using CellTiter-Glo® 2.0 assay that is based on the detection of the amount of adenosine triphosphate (ATP) generated primarily in the mitochondria to assess mitochondrial activity. The XTT and CellTiter-Glo® 2.0 assay are therefore used to assess cell viability and cell growth, with the XTT assay directly targeting mitochondrial activity, while the CellTiter-Glo® 2.0 assay indirectly reflects mitochondrial function by measuring ATP.

Although 2D models have many advantages in terms of user-friendliness, mechanistic manipulation, specific parameter measurement, cost-effectiveness, and time efficiency ﻿[72, 73], 2D models suffer from inherent limitations. Namely, they fail to capture the cellular heterogeneity and associated signaling cues linked to cell shape and orientation [74, 75]. This discrepancy can lead to inconsistencies with in vivo and clinical data, limiting the generalizability of results obtained from 2D models [76].

Many factors make 3D in vitro models a very valuable tool for cancer research because 3D in vitro models allow recapitulation of the tumor microenvironment [77] and provide cell-cell interactions, perfusion, and hypoxic conditions. To this end, cell proliferation, cell morphology and heterogeneity, cell migration, and gene expression are closer to in vivo in 3D in vitro models compared to 2D models [78, 79]. In the present study, the *beta-(1→3)(1→6)-glucan* from yeast could show only small effect on the proliferation of multicellular breast cancer tumor spheroids (Fig. 3) and the cytotoxicity of NK cells against MCTS of breast cancer cell lines (Supplementary Fig. 1) compared with the 2D model. This small effect of beta-glucan on 3D model compared to 2D model may justify that tumor cells in 3D models are usually more resistant to drugs and more invasive than in 2D models [76, 80–82].

## Conclusions

Yeast derived *beta-(1,3)(1,6)-D-glucan* could contribute to the treatment of cancer. It could increase the proliferation of primary NK cells (isolated from PBMC) in the presence and significantly in the absence of IL-2 (70 U/ml). Moreover, beta-glucan significantly inhibited the proliferation of breast cancer cell lines in 2D model at the highest beta glucan concentration (100 µg/ml), and the strongest inhibitory effect of beta glucan was on the MCF-7 cell line. Beta glucan also induced a weak, non-significant growth inhibitory effect on multicellular breast cancer tumor spheroids (3D). Furthermore, yeast *beta-(1→3)(1→6)-D-glucan* showed little effect on the proliferation of MCTS of breast cancer and cytotoxicity of NK cells against MCTS of breast cancer cell lines compared to the 2D model. These results suggest that beta-(1,3)(1,6)-D-glucan has an immunomodulatory and anticancer effect. Nevertheless, it is important to investigate other (higher) beta-glucan concentrations and the consequences of different beta-glucan sources on immune cells with antitumor activities, which may cause tumor regression and affect innate immunity, and to verify the actual clinical efficacy of beta-glucans.

### Supplementary Information

**Supplementary material 1.**

## Data Availability

The datasets used and/or analysed during the current study available from the corresponding author on reasonable request.

## Abbreviations

BC: Breast Cancer
BG: Beta glucan
CR3: Complement Receptor 3
DMEM: Dulbecco’s modified Eagle’s medium
FBS: Fetal Bovine Serum
IARC: International Agency for Research on Cancer
IL2: Interleukin 2
MAMP: Microbe-Associated Molecular Pattern
MCTS: Multicellular Tumor Spheroids
NK: cells Natural Killer cells
PAMP: Pathogen-Associated Molecular Patterns
PBMC: Peripheral Blood Mononuclear Cells
Pen/Strep: Penicillin/Streptomycin
PRRs: Pattern Recognition Receptors
ROS: Reactive oxygen species
RPMI: Roswell Park Memorial Institute
TLR: Toll-like receptors
XTT: 2,3-Bis-(2-Methoxy-4-Nitro-5-Sulfophenyl)-2 H-Tetrazolium-5-Carboxanilide
