# Supplementary material for: Role of beta-(1→3)(1→6)-D-glucan derived from yeast on natural killer (NK) cells and breast cancer cell lines in 2D and 3D cultures
Source: BMC Cancer. 2024 Mar 14;24:339. doi: 10.1186/s12885-024-11979-3 (PMC10938759; doi:10.1186/s12885-024-11979-3)
Supplement: Supplementary file 1 — Supplementary material 1. [file 12885_2024_11979_MOESM1_ESM.pdf]

Supplementary Figure

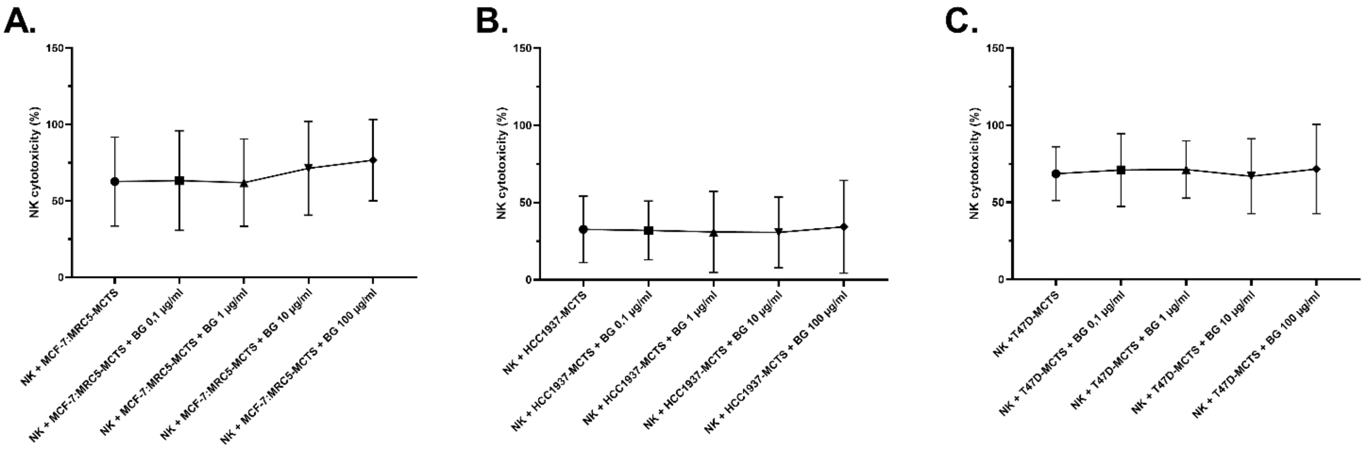

Supplementary Figure 1 │ Effect of *beta-(1→3)(1→6)-glucan* from yeast on the cytotoxicity of NK cells against the breast cancer Multicellular Tumor Spheroids (MCTS) (3D). The figure shows the percent variation of cytotoxicity of NK cells against (A) MCF-7 + MRC5 spheroids [1:1], (B) HCC1937 spheroids and (C) T47D spheroids with different β-glucan concentrations (0.1 - 1 - 10 and 100 µg/ml) after 72h incubation compared with untreated spheroids (control) at an E:T ratio of 3:1. The cytotoxicity was measured from the ATP released from the target and effector cells using CellTiter-Glo® 2.0 Assay. The results were pre-sented in three independent experiments. Statistical significance was determined by two-way ANOVA. * $p < 0.05$; ** $p < 0.01$; *** $p < 0.001$; **** $p < 0.0001$.
